# Simultaneous Inhibition of Three Major Cytokines and Its Therapeutic Effects: A Peptide-Based Novel Therapy against Endotoxemia in Mice

**DOI:** 10.3390/jpm11050436

**Published:** 2021-05-20

**Authors:** Hung-Jen Shih, Chao-Yuan Chang, Milton Chiang, Van Long Le, Hao-Jen Hsu, Chun-Jen Huang

**Affiliations:** 1 Department of Urology, School of Medicine, College of Medicine, Taipei Medical University, Taipei 110, Taiwan; jasta1206@tmu.edu.tw; 2 Department of Urology, Wan Fang Hospital, Taipei Medical University, Taipei 116, Taiwan; 3 Department of Urology, Changhua Christian Hospital, Changhua 500, Taiwan; 4 Department of Anesthesiology, Wan Fang Hospital, Taipei Medical University, Taipei 116, Taiwan; yuanc669@gmail.com; 5 Integrative Research Center for Critical Care, Wan Fang Hospital, Taipei Medical University, Taipei 116, Taiwan; 6 Graduate Institute of Clinical Medicine, College of Medicine, Taipei Medical University, Taipei 110, Taiwan; d142108016@tmu.edu.tw (M.C.); drlong.gmhshue@gmail.com (V.L.L.); 7 Department of Anesthesiology and Critical Care, Hue University of Medicine and Pharmacy, Hue City 52000, Vietnam; 8 Department of Life Sciences, College of Medicine, Tzu Chi University, Hualien 970, Taiwan; 9 Department of Anesthesiology, School of Medicine, College of Medicine, Taipei Medical University, Taipei 110, Taiwan

**Keywords:** TNF-α, IL-1β, IL-6, necroptosis, pyroptosis, apoptosis, liver

## Abstract

Three major cytokines, including tumor necrosis factor-α (TNF-α), interleukin-1β (IL-1β), and IL-6, mediate endotoxemia-induced liver injury. With the similar structures to the binding domains of the three cytokines to their cognate receptors, the novel peptide KCF18 can simultaneously inhibit TNF-α, IL-1β, and IL-6. We elucidated whether KCF18 can alleviate injury of liver in endotoxemic mice. Adult male mice (BALB/cJ) were intraperitoneally (i.p.) administered lipopolysaccharide (LPS, 15 mg/kg; LPS group) or LPS with KCF18 (LKCF group). Mice in the LKCF group received KCF18 (i.p.) at 2 h (0.6 mg/kg), 4 h (0.3 mg/kg), 6 h (0.3 mg/kg), and 8 h (0.3mg/kg) after LPS administration. Mice were sacrificed after receiving LPS for 24 h. Our results indicated that the binding levels of the three cytokines to their cognate receptors in liver tissues in the LKCF group were significantly lower than those in the LPS group (all *p* < 0.05). The liver injury level, as measured by performing functional and histological analyses and by determining the tissue water content and vascular permeability (all *p* < 0.05), was significantly lower in the LKCF group than in the LPS group. Similarly, the levels of inflammation (macrophage activation, cytokine upregulation, and leukocyte infiltration), oxidation, necroptosis, pyroptosis, and apoptosis (all *p* < 0.05) in liver tissues in the LKCF group were significantly lower than those in the LPS group. In conclusion, the KCF18 peptide–based simultaneous inhibition of TNF-α, IL-1β, and IL-6 can alleviate liver injury in mice with endotoxemia.

## 1. Introduction

Tumor necrosis factor-α (TNF-α), interleukin-1β (IL-1β), and IL-6 are three major inflammatory cytokines that actively participate in mediating the development of organ injury (e.g., that of the liver) induced by endotoxemia and sepsis [1,2,3]. The three major inflammatory cytokines are produced by infectious, stimulus-activated immune cells during endotoxemia and sepsis and released into circulation in the early phase of endotoxemia and sepsis through activation of Toll-like receptor followed by the subsequent activation of nuclear factor-κB (NF-κB) [4,5]. In experimental studies, TNF-α, IL-1β, and IL-6 peaked in concentration within 90, 120, and 240 min, respectively, after endotoxin infusion [6,7]. Moreover, clinical studies on patients with endotoxemia and sepsis have reported robust correlations of the three major inflammatory cytokines concentrations with disease severity and organ dysfunction or failure [8,9].

Through directly activating NF-κB to prompt inflammatory cytokines (e.g., IL-6) and chemokines upregulation, TNF-α plays a central role in mediating inflammatory response [10]. TNF receptor 1 (TNFR1) is a transmembrane death receptor and the cognate receptor of TNF-α [11]. When binding with circulating TNF-α, TNFR1 can provoke the cell death processes of necroptosis and apoptosis and cause damage to vital organs (e.g., liver) [11,12,13]. IL-1β also plays a central role in mediating inflammatory response [14]. Upon induction, IL-1β is firstly presented as the inactive precursor form pro-IL-1β [15]. Then, this inactive form pro-IL-1β will be transformed into the active form IL-1β following caspase-1-mediated cleavage [15]. With binding to its cognate receptor IL-1 receptor (IL-1R), IL-1β prompts MyD88 recruitment and NF-κB activation to cause subsequent productions of inflammatory cytokines (e.g., IL-6) and chemokines [14,16]. IL-1β is also associated with the cell death process of pyroptosis, which involves activation of the crucial nucleotide-binding oligomerization domain-like receptor protein 3 (NLRP3) inflammasome pathway and caspase-1 [15,17]. IL-6, similar to TNF-α and IL-1β, also plays a central role in mediating inflammatory response [18]. Acting through binding with IL-6 receptor (IL-6R), i.e., the cognate receptor, IL-6 exerts various pathological effects (e.g., vascular endothelial breakdown) that may result in organ injury (i.e., through classical IL-6 signaling) [19]. In addition to IL-6R, classical IL-6 signaling activates crucial pathways of STAT3 and Akt [20]. Moreover, the three major inflammatory cytokines can increase the production of reactive oxygen and nitrogen species and impose oxidative stress in vital organs [8]. Subsequently, oxidative stress may contribute to organ injury induced by endotoxemia and sepsis [8].

Anticytokine therapy that involves the blockade of cytokines and the inhibition of the binding of cytokines to their cognate receptors has been developed for treating endotoxemia and sepsis. For instance, we recently developed a novel peptide-based anti-TNF-α therapy against endotoxemia and confirmed its therapeutic effects [21]. Our results demonstrated that the novel peptide SEM18, with the structures similar to the binding domain of TNF-α to TNFR1, can act by binding with TNF-α (i.e., as a binding decoy) to, in turn, reduce TNF-α/TNFR1 binding, thus ameliorating organ injury and improving survival in endotoxemic mice [21]. In addition, the beneficial effects of monoclonal antibody-based anti-TNF-α, anti-IL-1β, or anti-IL-6 therapy individually on sepsis have previously been reported [22,23,24]. However, treatments that can simultaneously block the three major inflammatory cytokines and exert therapeutic effects on endotoxemia and sepsis remain to be developed.

A study employed a molecular docking simulation technique [25] to design a novel peptide KCF18 possessing structures similar to the binding domains of the three major inflammatory cytokines to the respective cognate receptors [26]. The findings in an ex vivo model demonstrated that KCF18 can act as a decoy for bindings to concurrently block TNF-α, IL-1β, and IL-6 [26]. Furthermore, the results in ex vivo experiments revealed the potent anti-inflammatory activity of KCF18 [26]. In this study, we elucidated the therapeutic effects of the novel peptide KCF18 on endotoxemia by using an endotoxin-induced monomicrobial sepsis murine model. We hypothesized that KCF18 can alleviate liver injury in endotoxin-treated mice. We determined whether KCF18 can reduce the bindings of TNF-α/TNFR1, IL-1β/IL-R, and IL-6/IL-6R in liver tissues. In addition, we elucidated possible underlying mechanisms in mediating therapeutic effects of the KCF18 peptide against endotoxemia-induced injury of liver, particularly cell death processes, namely necroptosis, pyroptosis, and apoptosis. Moreover, as demonstrated in our recent SEM18 peptide study, the in vivo half life of peptides was short [21]. Based on those data, this study designed a therapeutic regimen with a loading dose of peptide followed by repeated doses of peptide to achieve and maintain therapeutic levels.

## 2. Materials and Methods

### 2.1. Approval of Animal Experiment and Study

We used male, adult BALB/cJ mice (age: 7–8 weeks) from Taiwan National Laboratory Animal Center (Taipei, Taiwan) in this study. All mice were maintained on a 12 h light/12 h dark cycle and provided with standard laboratory mouse diet and accessing to water freely. The care and handling of the mice were conducted in conformity with the US National Institutes of Health (NIH) guidelines. The Institutional Animal Use and Care Committee, Taipei Medical University, approved all the animal studies (LAC-2017-0206).

### 2.2. Peptide Design and Synthesis

KCF18 (molecular weight: 2195.92 Da) based on structures derived from TNFR1, IL-1R, and IL-6R was designed using a protein docking ZDOCK software (Biovia Discovery Studio 3.5; Biovia, San Diego, CA, USA) per a method reported previously [26]. In addition, a random peptide (KCP; molecular weight: 2195.92 Da) was designed as the control. KCF18 and KCP were both synthesized (Mission Biotech, Taipei, Taiwan). High-performance liquid chromatography data revealed that the purity of both peptides was 95%.

### 2.3. Pharmacokinetic Analysis

For KCF18 pharmacokinetic analysis, a set of mice was employed. Pharmacokinetic analysis was performed according to the method of a previous study [27]. In brief, the mice were intraperitoneally (i.p.) administered one dose of KCF18 (0.6 mg/kg). Serial samples of blood were obtained through submandibular vein puncture before dosing (time 0) and at another 5 time points (i.e., 0.5, 1, 2, 4, 8, and 12 h) after KCF18 administration. Blood samples were centrifuged (3000 rpm, 10 min) immediately after collection. Then, plasma samples were collected and stored (−80 °C) until analysis. The plasma concentrations of KCF18 were analyzed through liquid chromatography–tandem mass spectrometry (Xevo TQ-S with Acquity UPLC pump system; Waters, Milford, MA, USA). Data were analyzed with MassLynx 4.1 Software (version 4.1, SCN 714; Waters) to facilitate the pharmacokinetic analysis of KCF18.

The KCF18 in vivo half life, as our data demonstrated, was estimated to be 2 h. Detailed information is provided in Section 3.1.

### 2.4. Ex Vivo Bioluminescence Imaging

The biodistribution of KCF18 was determined by performing ex vivo bioluminescence imaging analysis per a previously reported method [21]. For imaging, KCF18 was conjugated with rhodamine B (Abcam, Cambridge, UK). One dose (0.6 mg/kg, i.p.) of rhodamine B-conjugated KCF18 was injected. Two hours later, the mice were euthanized, and the organs (including heart, lung, liver, kidney, and spleen) were collected. An in vivo imaging system (IVIS Lumina XRMS; PerkinElmer, Waltham, MA, USA) was employed and bioluminescence imaging assay was conducted. Images were analyzed with PerkinElmer’s Living Image software.

### 2.5. Cytokine Receptor Binding Assay

Immunofluorescence staining and the proximity ligation assay (PLA) were performed to evaluate bindings of TNF-α, IL-1β, and IL-6 to TNFR1, IL-1R, and IL-6R, respectively, in liver tissues, per a previously reported method [28]. A DuoLink Mouse Rabbit in situ PLA kit from Sigma-Aldrich (St. Louis, MO, USA) was employed, and the assay was performed per the manufacturer’s protocol. After blocking, sections of liver tissues were incubated with primary antibodies against TNF-α and TNFR1, IL-1β and IL-1R, or IL-6 and IL-6R (all from Abcam, Cambridge, UK), followed by incubation with secondary antibodies (namely PLA probes) labeled with oligonucleotide. Nuclei staining was performed using 4′,6-diamidino-2-phenylindole (DAPI; Sigma-Aldrich). A microscope (DeltaVision Elite microscope; GE Healthcare, Marlborough, MA, USA) was employed for visualizing tissue sections. All images were analyzed with the image processing software Image J (a free software developed by NIH, USA; https://imagej.nih.gov/ij/, accessed on 11 July 2018.).

### 2.6. Endotoxemia Murine Model and Peptide Therapy

Gram (-) endotoxin (lipopolysaccharide, LPS, *Escherichia coli* 0127:B8 from Sigma-Aldrich) was employed to prompt endotoxemia and liver injury per a previously reported method [29]. The mice were randomly allocated to receive LPS (15 mg/kg, i.p.), LPS with KCF18, or LPS with KCP (designated as the LPS group, LKCF group, and LKCP group, respectively). To serve as the respective control of those groups treated with LPS, another set of mice was allocated randomly to receive 0.9% saline (NS; 0.5 mL, i.p.), NS with KCF18, or NS with KCP (designated as the Sham group, KCF group, and KCP group, respectively). Four doses of the peptide were administered to the mice receiving peptide therapy. The first dose (KCF18 or KCP, 0.6 mg/kg, i.p.) was injected 2 h after LPS or NS, and then, 3 supplemental doses (KCF18 or KCP, 0.3 mg/kg) were respectively injected 4, 6, and 8 h after LPS or NS, according to KCF18 pharmacokinetic data.

### 2.7. Blood Sampling, Liver Harvesting, and Wet/Dry Weight (W/D) Ratio Determination

All mice were closely monitored for 24 h. The surviving mice received zoletil/xylazine (40/10 mg/kg, i.p.) for anesthesia. After obtaining blood samples, decapitation was performed to euthanize the mice. Then, the liver tissues were collected. Part of the liver tissues (the right and median lobes) were immediately frozen with liquid nitrogen and then stored (−80 °C) for later analysis. Part of the liver tissues (the left lobe) was placed in 10% formalin (Sigma-Aldrich) for histological analysis.

Moreover, for liver water content assay, part of the liver tissues (the caudate lobe) was freshly collected, weighed, placed in an oven (80 °C) for 24 h, and weighed again. Then, the W/D weight ratio was calculated to determine liver water content [30].

### 2.8. Vacular Permeability Assay

Vascular permeability was examined using an Evans blue dye (EBD) extravasation assay [31]. A set of mice was treated per the aforementioned method. At 23 h after NS or LPS administration, EBD (2% solution in NS; 2 mL/kg, Sigma-Aldrich) was administered intravenously. One hour later, mice received zoletil/xylazine (40/10 mg/kg, i.p.) for anesthesia and then thoroughly perfused with NS to remove residual EBD. The liver tissues were harvested, weighed, and then homogenized (1:3 volume ratio of 50% trichloroacetic acid, Sigma-Aldrich). After the removal of tissue debris and protein precipitates, the EBD concentration in supernatants was determined through spectroscopy by examining absorbance at 620 nm.

### 2.9. Liver Enzyme Measurements

Liver enzymes concentrations, namely alanine aminotransferase (ALT) and aspartate aminotransferase (AST), in the collected blood samples were measured using the Vitros 750 autoanalyzer (Johnson & Johnson, New Brunswick, NJ, USA) to determine injury level of liver [21].

### 2.10. Histological Analysis of Liver

The liver tissues fixed in formalin were placed in paraffin wax, followed by being serially sectioned and then stained using hematoxylin and eosin. Histological characteristics, including infiltration of polymorphonuclear neutrophil (PMN), focal necrosis, interstitial edema, and congestion/hemorrhage, were appraised with a light microscope. Then, the liver injury level was determined by calculating Suzuki scores (namely sum scores of vacuolization (0: none; 4: severe), necrosis (0: none; 4: >60%), and congestion (0: none; 4: severe)) [32].

### 2.11. Enzyme-Linked Immunosorbent Assay (ELISA)

Hepatic concentrations of cytokines were assayed using ELISA. Commercial kits of ELISA for TNF-α, pro-IL-1β, and IL-6 (Enzo Life Science, Farmingdale, NY, USA) were used. Snap-frozen tissues were crushed (on dry ice), homogenized (at low speed with protease inhibitors), and centrifuged per the manufacturer’s protocol. Then, the supernatants were collected and ELISA was performed to determine the hepatic cytokines concentrations, also according to the manufacturer’s protocol.

### 2.12. Immunohistochemistry Staining

The statuses of lipid peroxidation, M1/M2 macrophage polarization, necroptosis, and pyroptosis were determined through immunohistochemistry staining, per a previously reported method [7,8,9,10,11]. Liver tissues (in paraffin sections) were incubated with primary antibodies against one of the following: malondialdehyde (MDA; Abcam), a lipid peroxidation-related protein [33]; inducible nitric oxide synthase (iNOS; Abcam), an M1 phase macrophage polarization-related protein [34]; CD206 (Abcam), an M2 phase macrophage polarization-related protein [34]; phosphorylated mixed lineage kinase domain-like pseudokinase (pMLKL; Abcam), a necroptosis-related protein [35]; or NLRP3 (Abcam), a pyroptosis-related protein [17]. All the sections were observed under the TissueGnostics Axio Observer Z1 microscope (TissueGnostics, Vienna, Austria) and then quantified and analyzed using the image processing software Image J.

### 2.13. The Terminal Deoxynucleotidyl Transferase dUTP Nick End Labeling (TUNEL) Assay

The key characteristic of apoptosis, i.e., DNA fragmentation, in liver tissues was examined using the TUNEL assay [36]. A kit for in situ cell death detection (Roche, Indianapolis, IN, USA) was used. Apoptotic cells were stained, as per the manufacturer’s protocol. In addition, DAPI staining (Sigma-Aldrich) was conducted to examine the nuclei. After scanning, five random fields (0.25 mm^2^) were selected to facilitate calculating the mean TUNEL-positive cells of each group.

### 2.14. Immunoblotting Assay

Protein extraction from the freshly frozen liver tissues were conducted per a previously described method [21]. Then, proteins from each group (100 μg) were segregated via electrophoresis and passed onto nitrocellulose membranes (Bio-Rad Laboratories, Hercules, CA, USA). The membranes were subsequently incubated with one of the following primary antibodies: the anti-phospho-Akt (Thr308) antibody (p-Akt, #2965, Cell Signaling Technology, Danvers, MA USA), anti-Akt antibody (#4691S, Cell Signaling), anti-phospho-STAT3 (Thr705) antibody (p-STAT3, #9145, Cell Signaling), anti-STAT3 antibody (#30835, Cell Signaling), anti-caspase-8 (EPR17367) antibody (ab1844721, Abcam), anti-caspase-1 (EPR4321) antibody (ab108362, Abcam), anti-BAX antibody (ab32503, Abcam), anti-Bcl-2 antibody (#2870, Cell Signaling), anti-actin antibody (A5441, Sigma-Aldrich; as an internal standard), and anti-tubulin antibody (a7291, Abcam; also as an internal standard). Then, the membranes were incubated with horseradish peroxidase-conjugated secondary antibody (Abcam). Bound antibody was recognized using chemiluminescence (ECL Plus kit; Amersham, Buckinghamshire, UK). Then, the membranes were scanned (Invitrogen iBright CL1500 Imaging Systems, Thermo-Fischer, Waltham, MA, USA). The density of each protein band on the scanned digital images was measured using the image processing software Image J for densitometry analysis. Then, each protein band density was compared to that of the internal standard (actin or tubulin) for normalization. The relative BAX/Bcl-2 ratio, which indicates susceptibility to apoptosis [21], was also calculated.

### 2.15. Statistical Analysis

Data were calculated to determine the mean ± standard deviation. One-way analysis of variance with post hoc pairwise comparisons using Tukey’s test were performed to analyze differences between groups. A *p* value of <0.05 was considered as significant. The software SPSS v21.0 (SPSS, Somers, NY, USA) was employed for statistical analysis.

## 3. Results

### 3.1. Pharmacokinetics and Tissue Distribution of KCF18

**Figure 1:**
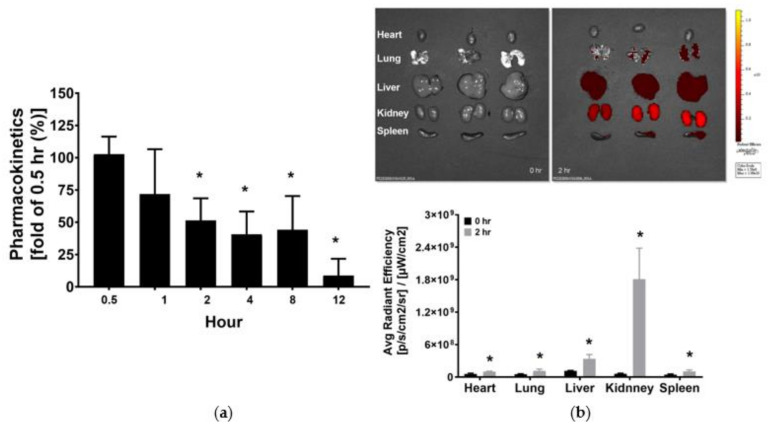
(**a**) Pharmacokinetic analysis of KCF18. The plasma concentrations of KCF18 were measured through the assay of liquid chromatography–tandem mass spectrometry. The KCF concentration measured 0.5 h after intraperitoneal (i.p.) administration was used as the baseline. (**b**) Biodistribution of KCF18. The fluorescence signal intensities of KCF18 were measured 0 h (baseline) and 2 h after i.p. administration through the assay of the ex vivo bioluminescence imaging method. Data regarding pharmacokinetics and biodistribution were obtained from four and three mice from each time point, respectively. * *p* < 0.05 relative to the baseline.

Figure 1a illustrates the pharmacokinetic data of KCF18. The plasma KCF18 concentration measured 1 h after KCF18 administration was not significantly different from that measured 0.5 h after KCF18 administration (i.e., the baseline). Notably, the plasma KCF18 concentrations measured 2, 4, and 8 h after KCF18 administration were comparable, and all were significantly lower than the baseline (approximately 50% of the baseline; *p* = 0.015, 0.003 and 0.006, respectively). Moreover, the mean plasma KCF18 concentration measured 12 h after KCF18 administration was low. Thus, the KCF18 in vivo half life was estimated to be 2 h after its i.p. administration.

As illustrated in Figure 1b, significant fluorescence signals of KCF18 were detected in the heart, lung, liver, kidney, and spleen 2 h after i.p. KCF18 administration. Furthermore, the signal intensities of KCF18 measured 2 h after KCF18 administration in the heart, lung, liver, kidney, and spleen were all significantly higher than the baseline (i.e., 0 h after i.p. KCF18 administration; *p* = 0.037, 0.046, 0.032, 0.032, and 0.030, respectively). These results demonstrated significant KCF18 distribution in major organs after its i.p. administration.

### 3.2. KCF18 Inhibits LPS-Induced Cytokine Receptor Bindings in the Liver

**Figure 2:**
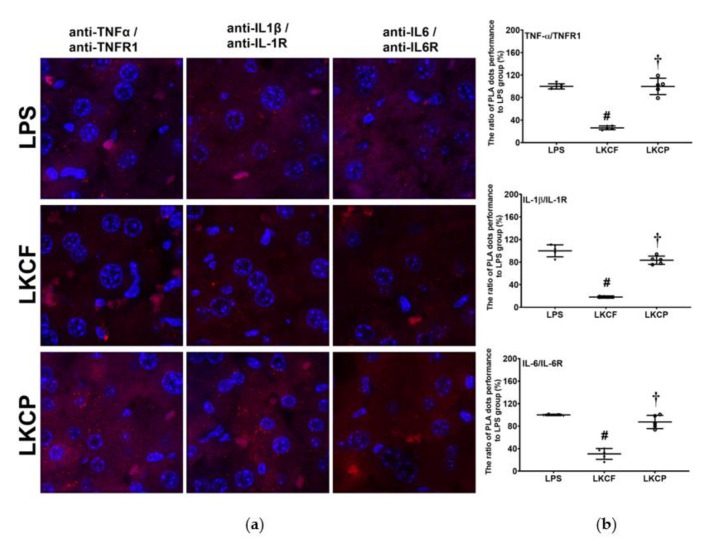
Binding assays of tumor necrosis factor-α (TNF-α)/TNF receptor 1 (TNFR1), interleukin-1β (IL-1β)/IL-1 receptor (IL-1R), and IL-6/IL-6R in liver tissues that was measured 24 h after intraperitoneal injection of lipopolysaccharide (LPS), using methods of immunofluorescence staining and the proximity ligation assay (PLA). (**a**) Characteristic microscopic images of PLA. A positive PLA signal was identified as red dots, indicating positive TNF-α/TNFR1, IL-1β/IL-1R, or IL-6/IL-6R protein/protein interactions in liver tissues, respectively. DAPI staining of nuclei in liver tissues was identified as blue dots. (**b**). The ratios of PLA dots, comparing to those in the LPS group. LPS: the LPS (15 mg/kg) group. LKCF: the LPS with KCF18 peptide group. LKCP: the LPS with control peptide group. Data are expressed as mean ± standard deviation. PLA data were derived from five mice from each group. ^#^ *p* < 0.05, LKCF group comparing to LPS group. ^†^ *p* < 0.05, LKCP group comparing to LKCF group.

Figure 2 illustrates the results of the binding assay performed to examine the binding of cytokines to their cognate receptors in liver tissues. No significant hepatic PLA signals of TNF-α/TNFR1, IL-1β/IL-1R, and IL-6/IL-6R were observed in the Sham, KCF, and KCP groups (data not shown). By contrast, significant hepatic PLA signals of TNF-α/TNFR1, IL-1β/IL-1R, and IL-6/IL-6R were noted in the LPS, LKCF, and LKCP groups (Figure 2a). The hepatic PLA signal intensities of TNF-α/TNF, IL-1β/IL-1R, and IL-6/IL-6R in the LKCF group were approximately 26.3%, 18.2%, and 30.7% of those in the LPS group, respectively. Our analyses revealed that the hepatic PLA signal intensities of TNF-α/TNFR1, IL-1β/IL-1R, and IL-6/IL-6R in the LKCF group were significantly lower than those in the LPS group (all *p* < 0.001, Figure 2b). Similar pictures were observed between the LKCF and the LKCP groups (all *p* < 0.001, Figure 2b). These results indicated that KCF18 could simultaneously inhibit bindings of the three major cytokines to their cognate receptors in liver tissues.

### 3.3. KCF18 Inhibits LPS-Induced Liver Injury and Oxidation

**Figure 3:**
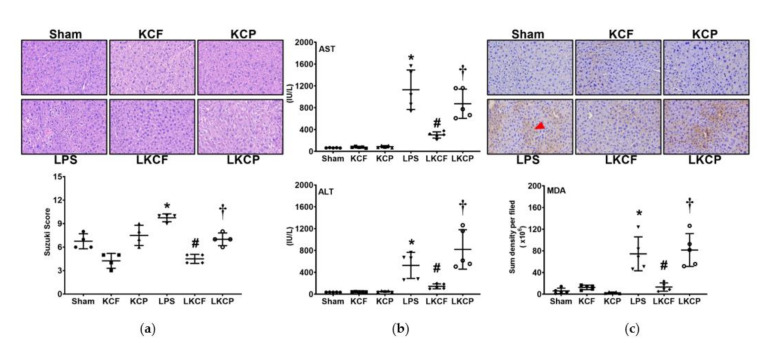
Liver injury and oxidative status. (**a**) Characteristic microscopic images of liver tissues with hematoxylin and eosin staining (200×) and Suzuki scores, i.e., liver injury scores. (**b**) Concentrations of aspartate aminotransferase (AST) and alanine aminotransferase (ALT) in plasma. (**c**) Characteristic microscopic images of malondialdehyde (MDA, as marked by the red arrow; 200×) in liver tissues determined with immunohistochemistry analysis as well as the sum of the quantitative intensity of MDA. Sham: the normal saline (NS) group. KCF: the NS with KCF18 peptide group. KCP: the NS with control peptide (CP) group. LPS: the lipopolysaccharide (LPS; 15 mg/kg) group. LKCF: the LPS with KCF18 peptide group. LKCP: the LPS with CP group. All data were measured 24 h after NS or LPS administration. Data are expressed as mean ± standard deviation. Data regarding Suzuki scores, AST, ALT, and MDA were derived from four, five, five, and five mice from each group, respectively. * *p* < 0.05, LPS group comparing to Sham group. ^#^ *p* < 0.05, LKCF group comparing to LPS group. ^†^ *p* < 0.05, LKCP group comparing to LKCF group.

All mice survived the experiment. Figure 3a shows the histological characteristics and injury scores (Suzuki scores) of the liver tissues. Liver injury was determined through measuring the AST and ALT concentrations in plasma (Figure 3b). Figure 3c presents the oxidation status determined by measuring the lipid peroxidation of the liver tissues. Histological analysis revealed no significant liver injury characteristics in the Sham, KCF, and KCP groups. Suzuki scores, plasma AST and ALT concentrations, and hepatic MDA expression levels were low in the Sham, KCF, and KCP groups. By contrast, histological analysis revealed significant characteristics of liver injury in the LPS group. Suzuki scores, AST and ALT concentrations in plasma, and hepatic MDA expression levels were all significantly higher in the LPS group than those in the Sham group (*p* = 0.002, < 0.001, = 0.003, and < 0.001, respectively). By contrast, the level of liver injury in the LKCF group was significantly lower than that in the LPS group. Our findings revealed lower Suzuki scores, plasma AST and ALT concentrations, and hepatic MDA expression levels in the LKCF group than those in the LPS group (*p* < 0.001, < 0.001, = 0.024, and < 0.001, respectively). Similar pictures were observed between the LKCF and the LKCP groups (Suzuki scores: *p* = 0.010; AST: *p* = 0.001; ALT: *p* < 0.001; MDA: *p* < 0.001). These results demonstrated that KCF18 alleviated LPS-induced liver injury and oxidation.

### 3.4. KCF18 Inhibits the LPS-Induced Increase in Vascular Permeability and Akt and STAT3 Activation

**Figure 4:**
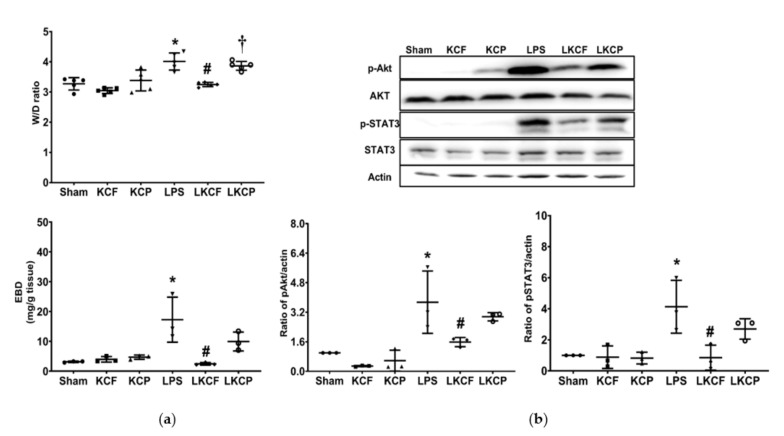
Vascular permeability status and activation levels of Akt and STAT3. (**a**) Wet/dry (W/D) weight ratio and Evans blue dye (EBD) concentration in liver tissues. (**b**) Characteristic gel images of phosphorylated Akt (p-Akt), Akt, phosphorylated STAT3 (p-STAT3), STAT3, and actin (as the internal standard) in liver tissues analyzed through immunoblotting assay; findings are presented with the relative band density of p-Akt/actin and p-STAT3/actin. Sham: the normal saline (NS) group. KCF: the NS with KCF18 peptide group. KCP: the NS with control peptide (CP) group. LPS: the lipopolysaccharide (LPS; 15 mg/kg) group. LKCF: the LPS with KCF18 peptide group. LKCP: the LPS with CP group. All data were measured 24 h after NS or LPS administration. Data are expressed as mean ± standard deviation. Data regarding the W/D weight ratio, EBD assay, p-Akt/Akt/actin, and p-STAT3/STAT3/actin were obtained from five, three, three, and three mice from each group, respectively. * *p* < 0.05, LPS group comparing to the Sham group. ^#^ *p* < 0.05, LKCF group comparing to the LPS group. ^†^ *p* < 0.05, LKCP group comparing to the LKCF group.

Figure 4a shows the findings of vascular permeability, as measured by calculating the W/D weight ratio and EBD concentration in liver tissues. Figure 4b shows the levels of Akt and STAT3 activation in the liver tissues, as measured through immunoblotting assay. Analyses of the Sham, KCF, and KCP groups revealed low W/D weight ratios, low EBD concentrations in liver tissues, and low hepatic concentrations of p-Akt and p-STAT3. By contrast, the W/D weight ratios, EBD concentrations in liver tissues, and hepatic concentrations of p-Akt and p-STAT3 were significantly higher in the LPS group than those in the Sham group (*p* < 0.001, = 0.003, = 0.034, and = 0.007, respectively). Notably, the W/D weight ratio and EBD concentrations in liver tissues were significantly lower in the LKCF group than those in the LPS group (*p* < 0.001 and = 0.003, respectively). In addition, the W/D weight ratio was significantly lower in the LKCF group than that in the LKCP group (*p* = 0.002). Moreover, the concentration of EBD, p-Akt, and p-STAT3 in the liver tissues were lower in the LKCF group than those in the LKCP group; however, the differences were not statistically significant (*p* = 0.15, 0.27, and 0.18, respectively). These findings demonstrated that KCF18 mitigated the LPS-induced increase in vascular permeability and Akt and STAT3 activation in the liver tissues.

### 3.5. KCF18 Inhibits LPS-Induced Liver Inflammation

**Figure 5:**
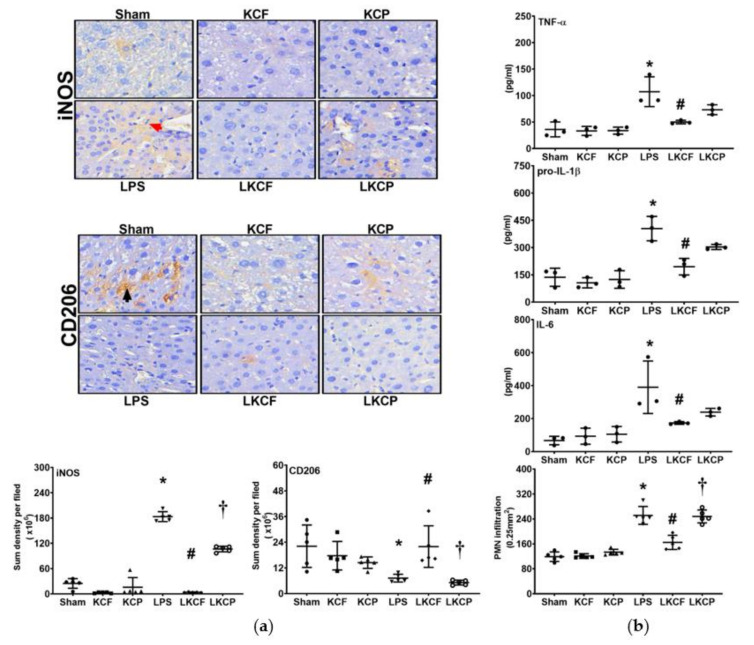
Liver inflammation status. (**a**) Characteristic microscopic images (200×) of inducible nitric oxide synthase (iNOS, as marked by the red arrow; the M1 phase polarization marker) and CD206 (as marked by the black arrow; the M2 phase polarization marker) in liver tissues obtained by performing immunohistochemistry analysis; findings are presented with the respective quantitative sum intensity of iNOS and CD206. (**b**) Concentrations of tumor necrosis factor-α (TNF-α), pro-interleukin-1β (pro-IL-1β), and interleukin (IL-6) in liver tissues, as determined using the enzyme-linked immunosorbent assay, and the polymorphonuclear leukocyte (PMN) infiltration level in liver tissues. Sham: the normal saline (NS) group. KCF: the NS with KCF18 peptide group. KCP: the NS with control peptide (CP) group. LPS: the lipopolysaccharide (LPS; 15 mg/kg) group. LKCF: the LPS with KCF18 peptide group. LKCP: the LPS with CP group. All data were measured 24 h after NS or LPS administration. Data are expressed as mean ± standard deviation. Data regarding iNOS, C206, TNF-α, pro-IL-1β, IL-6, and PMN infiltration were derived from 5, 5, 3, 3, 3, and 5 mice from each group, respectively. * *p* < 0.05, LPS group comparing to Sham group. ^#^ *p* < 0.05, LKCF group comparing to LPS group. ^†^ *p* < 0.05, LKCP group comparing to LKCF group.

Liver inflammation was determined by examining macrophage activation in the liver tissues (Figure 5a). The expression level of iNOS (i.e., M1 phase polarization) was low in the Sham, KCF, and KCP groups. By contrast, the iNOS expression level in the LPS group was significantly higher than that in the Sham group (*p* < 0.001). In addition, the iNOS expression level in the LKCF group was significantly lower than that in the LPS group (*p* < 0.001). Similarly, the iNOS expression level in the LKCF group was significantly lower than that in the LKCP group (*p* < 0.001). The expression level of CD206 (i.e., M2 phase polarization) was similar in the Sham, KCF, and KCP groups. In contrast to that of iNOS, the expression level of CD206 in the LPS group was significantly lower than that in the Sham group (*p* = 0.014). Moreover, the expression level of CD206 in the LKCF group was significantly higher than those in the LPS group (*p* = 0.014) and the LKCP group (*p* = 0.015). Liver inflammation was also determined by examining cytokine upregulation and PMN infiltration in the liver tissues (Figure 5b). Our findings revealed that the data of hepatic TNF-α, pro-IL-1β, and IL-6 concentrations as well as the data of PMN infiltration level paralleled the data of iNOS, except that the differences in hepatic TNF-α, pro-IL-1β, and IL-6 concentrations between the LKCF and the LKCP groups were not statistically significant (*p* = 0.389, 0.102, and 0.867, respectively). Collectively, these findings demonstrated that KCF18 alleviated LPS-induced liver inflammation.

### 3.6. KCF18 Inhibits LPS-Induced Liver Necroptosis

**Figure 6:**
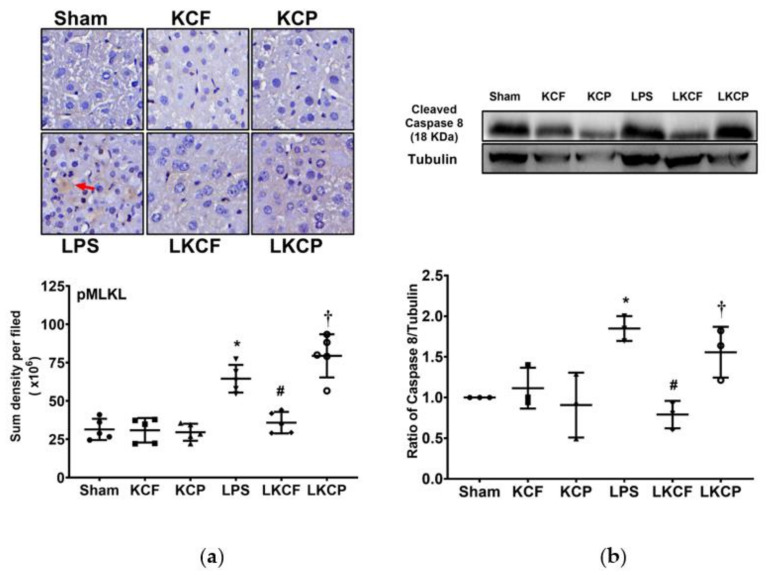
Liver necroptosis status. (**a**) Characteristic microscopic images (200×) of phosphorylated mixed lineage kinase domain-like pseudokinase (pMLKL, as marked by the red arrow) in liver tissues obtained by performing immunohistochemistry analysis; findings are presented with the sum quantitative intensity of pMLKL. (**b**) Characteristic gel images of cleaved caspase 8 and tubulin (as the internal standard) in liver tissues obtained through immunoblotting assay; findings are presented with the relative band density of cleaved caspase-8 and tubulin. Sham: the normal saline (NS) group. KCF: the NS with KCF18 peptide group. KCP: the NS with control peptide (CP) group. LPS: the lipopolysaccharide (LPS; 15 mg/kg) group. LKCF: the LPS with KCF18 peptide group. LKCP: the LPS with CP group. All data were measured 24 h after NS or LPS administration. Data are expressed as mean ± standard deviation. Data regarding pMLKL and cleaved caspase 8 were derived from five and three mice from each group, respectively. * *p* < 0.05, LPS group compared to the Sham group. ^#^ *p* < 0.05, LKCF group compared to the LPS group. ^†^ *p* < 0.05, LKCP group compared to the LKCF group.

Liver necroptosis status was examined by determining the expression levels of pMLKL and cleaved caspase 8 in the liver tissues (Figure 6a,b, respectively). The expression levels of pMLKL and cleaved caspase 8 were low in the Sham, KCF, and KCP groups. Moreover, the expression levels of pMLKL and cleaved caspase 8 in the LPS group were significantly higher than those in the Sham group (*p* < 0.001 and = 0.013, respectively). Notably, the expression levels of pMLKL and cleaved caspase 8 in the LKCF group were significantly lower than those in the LPS group (*p* < 0.001 and = 0.002, respectively) and those in the LKCP group (*p* < 0.001 and = 0.025, respectively). These data indicated that KCF18 could inhibit LPS-induced liver necroptosis.

### 3.7. KCF18 Inhibits LPS-Induced Liver Pyroptosis

**Figure 7:**
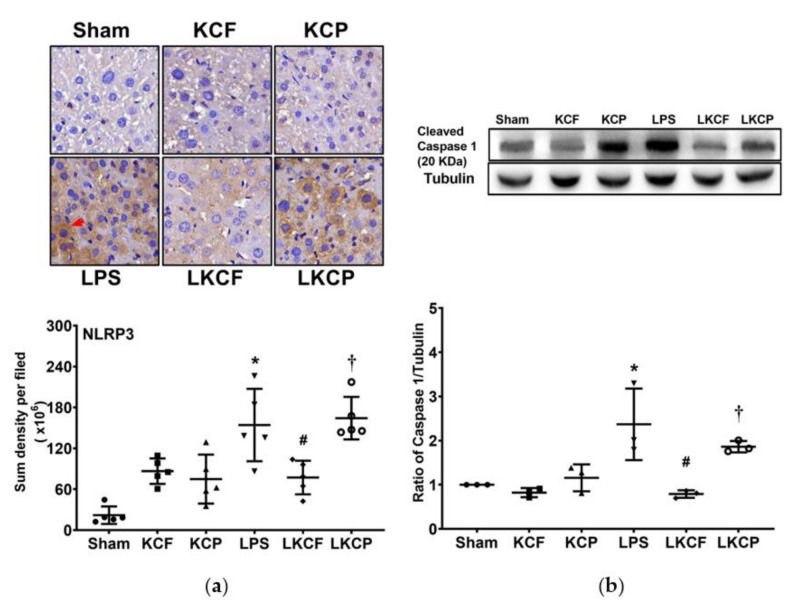
Liver pyroptosis status. (**a**) Characteristic microscopic images (200×) of nucleotide-binding oligomerization domain-like receptor protein 3 (NLRP3, as marked by the red arrow) in liver tissues obtained by performing immunohistochemistry analysis; findings are presented with the sum quantitative intensity of NLRP3. (**b**) Characteristic gel images of cleaved caspase 1 and tubulin (as the internal standard) in liver tissues obtained through immunoblotting assay; findings are presented with the relative band density of cleaved caspase-1 and tubulin. Sham: the normal saline (NS) group. KCF: the NS with KCF18 peptide group. KCP: the NS with control peptide (CP) group. LPS: the lipopolysaccharide (LPS; 15 mg/kg) group. LKCF: the LPS with KCF18 peptide group. LKCP: the LPS with CP group. All data were measured 24 h after NS or LPS administration. Data are expressed as mean ± standard deviation. Data regarding pMLKL and cleaved caspase 8 were derived from five and three mice from each group, respectively. * *p* < 0.05, LPS group compared to the Sham group. ^#^ *p* < 0.05, LKCF group compared to the LPS group. ^†^ *p* < 0.05, LKCP group compared to the LKCF group.

Liver pyroptosis status was examined by determining NLRP3 and cleaved caspase 1 expression levels in liver tissues (Figure 7a,b, respectively). Hepatic NLRP3 inflammasome and cleaved caspase 1 expression levels in the Sham, KCF, and KCP groups were low. By contrast, hepatic NLRP3 and cleaved caspase 1 expression levels in the LPS group were significantly higher than those in the Sham group (*p* < 0.001 and = 0.006, respectively). In addition, hepatic NLRP3 and cleaved caspase 1 expression levels in the LKCF group were significantly lower than those in the LPS group (*p* = 0.010 and = 0.002, respectively) and those in the LKCP group (*p* = 0.003 and = 0.032, respectively). These findings demonstrated that KCF18 could inhibit LPS-induced liver pyroptosis.

### 3.8. KCF18 Inhibits LPS-Induced Liver Apoptosis

**Figure 8:**
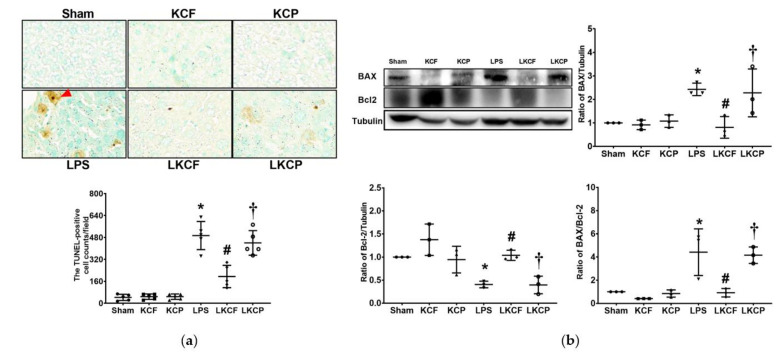
Liver apoptosis status. (**a**) Characteristic DNA fragmentation microscopic images (as marked by the red arrow) in liver tissues (200×) obtained through the terminal deoxynucleotidyl transferase dUTP nick end labeling (TUNEL) method and the count of TUNEL-positive cells (0.25 mm^2^). (**b**) Characteristic gel images of the proapoptotic BAX, the antiapoptotic Bcl-2, and tubulin (as the internal standard) in liver tissues obtained through immunoblotting assay as well as the band densities of BAX/tubulin, Bcl-2/tubulin, and BAX/Bcl-2 ratios. Sham: the normal saline (NS) group. KCF: the NS with KCF18 peptide group. KCP: the NS with control peptide (CP) group. LPS: the lipopolysaccharide (LPS; 15 mg/kg) group. LKCF: the LPS with KCF18 peptide group. LKCP: the LPS with CP group. All data were measured 24 h after NS or LPS administration. Data are expressed as mean ± standard deviation. Data regarding TUNEL, BAX, and Bcl-2 were derived from five, three, and three mice from each group, respectively. * *p* < 0.05, LPS group compared to the Sham group. ^#^ *p* < 0.05, LKCF group compared to the LPS group. ^†^ *p* < 0.05, LKCP group compared to the LKCF group.

Liver apoptosis status was determined by examining DNA fragmentation using the TUNEL assay (Figure 8a) and evaluating the expression levels of proapoptotic BAX and antiapoptotic Bcl-2 (Figure 8b) in the liver tissues. The counts of TUNEL-positive cells in the Sham, KCF, and KCP groups were low. By contrast, the count of TUNEL-positive cells in the LPS group was significantly higher than that in the Sham group (*p* < 0.001). The count of TUNEL-positive cells in the LKCF group was significantly lower than those in the LPS and LKCP groups (both *p* < 0.001). Similarly, the expression level of BAX in the Sham, KCF, and KCP groups was low. By contrast, the expression level of BAX in the LPS group was significantly higher than that in the Sham group (*p* = 0.035). Furthermore, the expression level of BAX in the LKCF group was significantly lower than those in the LPS and LKCP groups (*p* = 0.015 and = 0.029, respectively). The expression levels of Bcl-2 in the Sham, KCF, and KCP groups were comparable. By contrast, the Bcl-2 expression level in the LPS group was significantly lower than that in the Sham group (*p* = 0.037). Furthermore, the Bcl-2 expression level in the LKCF group was significantly higher than those in the LPS and LKCP groups (*p* = 0.025 and 0.022, respectively). Moreover, the findings for the BAX/Bcl-2 ratio were similar to those for BAX. These data indicated that KCF18 could inhibit LPS-induced liver apoptosis.

## 4. Discussion

**Figure 9:**
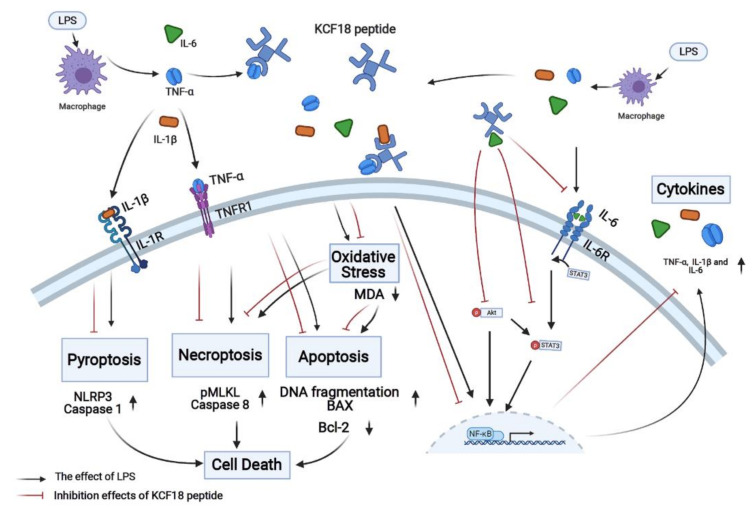
Diagram of the mechanisms of the novel peptide KCF18 on mitigating endotoxin-induced liver injury in mice. IL-1β, interleukin-1β. IL-1R: interleukin-1 receptor. IL-6: interleukin-6. IL-6R: interleukin-6 receptor. LPS: lipopolysaccharide. MDA: myeloperoxidase. NF-κB: nuclear factor-κB. NLRP3: nucleotide-binding oligomerization domain-like receptor protein 3. pMLKL: phosphorylated mixed lineage kinase domain-like pseudokinase. TNF-α: tumor necrosis factor-α. TNFR1: tumor necrosis factor receptor 1.

The novel peptide KCF18 was designed to act as a binding decoy to concurrently block TNF-α, IL-1β, and IL-6 and inhibit their bindings to their cognate receptors [26]. In the present study, the PLA data indicated that KCF18 could simultaneously inhibit more than 70% binding of TNF-α to TNFR1, more than 80% binding of IL-1β to IL-1R, and approximately 70% binding of IL-6 to IL-6R in the liver tissues of mice with endotoxemia. Considering that TNF-α, IL-1β, and IL-6 as well as their bindings to the cognate receptors play crucial roles in mediating the development of endotoxemia-induced organ injury, we hypothesized that KCF18 can alleviate acute liver injury in mice with endotoxemia. The findings of the present study confirmed our hypothesis and demonstrated that the novel peptide KCF18 could alleviate acute liver injury in the mice with endotoxemia. In addition, our results demonstrated that KCF18 could alleviate inflammation, oxidation, and cell death processes, namely necroptosis, apoptosis, and pyroptosis, in the liver tissues of mice with endotoxemia. Collectively, these findings confirmed the therapeutic potential of KCF18 against endotoxemia. The aforementioned mechanisms are summarized in Figure 9. Since effective therapies against endotoxemia and sepsis have not yet been developed, the findings of this study have potentially profound clinical implications and warrant further investigation.

TNF-α and IL-1β can activate NF-κB and induce inflammatory cytokine production (e.g., TNF-α, pro-IL-1β, and IL-6) [4,5,16]. KCF18 likely inhibits NF-κB activation and in turn suppresses endotoxin-induced cytokine production by blocking TNF-α and IL-1β. This hypothesis is supported by our finding that the hepatic TNF-α, pro-IL-1β, and IL-6 concentrations were significantly lower in the endotoxemic mice that were administered KCF18 than in the endotoxemic mice that were not administered KCF18. Behaving in line with this hypothesis, the endotoxemic mice that were administered KCF18 tended to exhibit M2 macrophage polarization in their liver tissues, whereas the endotoxemic mice that were not administered KCF18 tended to exhibit M1 macrophage polarization in the liver tissues. These findings demonstrated that KCF18 could inhibit macrophage activation in the endotoxemic mice. In addition, our findings demonstrated that KCF18 could inhibit leukocyte infiltration in the liver tissues of the endotoxemic mice. Collectively, these findings demonstrated that KCF18 alleviates endotoxemia-induced liver injury by inhibiting the inflammatory response.

The results of this study demonstrated that KCF18 suppressed endotoxemia-induced lipid peroxidation in the liver tissues. ROS and RNS induced by oxidative stress may cause tissue damage [37], and cytokines of TNF-α, IL-1β, and IL-6 can increase the production of ROS and RNS during endotoxemia [8,9]. Notably, iNOS is the main source of RNS (e.g., NO) during endotoxemia, and iNOS expression is tightly regulated by NF-κB [38]. KCF18 likely inhibits the activation of NF-κB by blocking TNF-α as well as IL-1β. Consistent with the aforementioned description, our findings demonstrated that hepatic iNOS expression was significantly lower in the endotoxemic mice that were administered KCF18 than that in the endotoxemic mice that were not administered KCF18. Collectively, these data suggested that KCF18 inhibits endotoxemia-induced oxidative stress partly by inhibiting iNOS upregulation in the liver tissues of endotoxemic mice.

Vascular endothelial breakdown triggered by upregulated IL-6 and its subsequent binding to IL-6R (i.e., the classical IL-6 signaling) contributes to endotoxemia-induced organ injury [19]. Our findings demonstrated that the levels of vascular permeability and water content were lower in liver tissues of the endotoxemic mice administered KCF18 than in those of the endotoxemic mice not administered KCF18. These findings indicated that KCF18 could restore vascular endothelial integrity in the liver tissues of the endotoxemic mice. Classical IL-6 signaling involves STAT3 and PI3K/Akt pathway activations [20]. Our immunoblotting results revealed that the activation levels of STAT3 and Akt in liver tissues were both lower in the endotoxemic mice administered KCF18 than in the endotoxemic mice not administered KCF18. These findings indicated that KCF18 exerts its therapeutic effects on endotoxemia-induced liver injury by modulating classical IL-6 signaling, inhibiting STAT3 and Akt activation as well as restoring vascular endothelial integrity.

Crucial mechanisms mediating endotoxemia-induced acute organ injury involve cell death processes, including necroptosis, pyroptosis, and apoptosis [11,12,13,15,17]. Upon binding with TNF-α, the transmembrane death receptor TNFR1 can prompt cell death processes of necroptosis and apoptosis [11,12,13]. Moreover, IL-1β upregulation is associated with pyroptosis [15,17]. The findings of this study revealed the significant upregulation of necroptosis-related proteins (i.e., MLKL and caspase-8) [35] and pyroptosis-related proteins (i.e., NLRP3 and caspase-1) [17] in the liver tissues of the entotoxemic mice. In addition, our findings revealed a significant increase in the count of TUNEL-positive cells, the upregulation of the proapoptotic protein BAX, the downregulation of the antiapoptotic protein Bcl-2, and an increase in the BAX/Bcl-2 ratio [36] in the liver tissues of the endotoxemic mice. These findings indicated the occurrence of significant necroptosis, pyroptosis, and apoptosis in the liver tissues of endotoxemic mice. Furthermore, our results revealed lower expression levels of pMLKL, caspase-8, NLRP3, caspase-1, and BAX; a lower count of TUNEL-positive cells; and a lower BAX/Bcl-2 ratio in the liver tissues of endotoxemic mice administered KCF18 than in those of the endotoxemic mice not administered KCF18. By contrast, we observed a higher expression level of the antiapoptotic Bcl-2 in the liver tissues of endotoxemic mice administered KCF18 than in those of the endotoxemic mice not administered KCF18. These findings indicated that KCF18 could inhibit endotoxemia-induced necroptosis, pyroptosis, and apoptosis in liver tissues. Collectively, these results demonstrated that KCF18 exerted its therapeutic effects on endotoxemia-induced liver injury by inhibiting necroptosis, pyroptosis, and apoptosis.

All existing anticytokine therapies against sepsis, including monoclonal antibody-based anticytokine therapies and our recently developed SEM18 peptide-based anti-TNF-α therapy, were designed to target a single cytokine and thus can inhibit only one cytokine when administered to treat endotoxemia and sepsis. Since the novel peptide KCF18 could simultaneously inhibit three cytokines (namely TNF-α, IL-1β, and IL-6), we conducted this study to determine whether the simultaneous inhibition of TNF-α, IL-1β, and IL-6 by the KCF18 peptide could exert beneficial effects against endotoxemia (i.e., a model of monomicrobial sepsis). In accordance with previous ex vivo data, the results of this study indicated that KCF18 could simultaneously inhibit TNF-α, IL-1β, and IL-6 and also inhibit their bindings to TNFR1, IL-1R, and IL-6R, respectively. Furthermore, the findings of the present study indicated that KCF18 could alleviate acute liver injury in the endotoxemic mice. These results confirmed the potent therapeutic effects of KCF18 against endotoxemia. Moreover, the findings of this study supported the concept that the KCF18 peptide-based concurrent inhibition of three cytokines can be a novel therapeutic strategy against endotoxemia and sepsis. Moreover, the KCF18 peptide-based therapy has several advantages over the existing anticytokine therapies against endotoxemia and sepsis. First, the KCF18 peptide-based therapy could simultaneously inhibit three cytokines. However, unlike the KCF18 peptide-based therapy, existing anticytokine therapies can inhibit only a single cytokine. Thus, three anticytokine therapies would be required to achieve the same effect exerted by the KCF18 peptide-based therapy. Therefore, this KCF18 peptide-based therapy is simpler and more feasible for clinical application compared with existing anticytokine therapies. Second, most of the existing anticytokine therapies employ monoclonal antibodies to inhibit cytokines. However, monoclonal antibodies are expensive. Moreover, developing a monoclonal antibody, either using the hybridoma or phage display technique [39,40], is considerably more expensive in time, labor, and money [22,41]. By comparison, a peptide is cheaper [21,26]. A peptide can be designed and synthesized through molecular docking simulation [25]. In this technique, a small molecule representing the crucial part of a target structure is generated by a computer after a series of simulations with various positions, conformations, and orientations [25]. With the use of molecular docking simulation, the development of a peptide and its subsequent modification is quicker and cheaper [25]. Moreover, monoclonal antibodies are large molecules that can exert some side effects (e.g., anaphylaxis) [22]. By contrast, peptides are small molecules that are relatively safe and well tolerated [21,42]. Therefore, this KCF18 peptide-based therapy can be a cheaper and safer treatment approach against endotoxemia and sepsis compared with existing monoclonal antibody-based anticytokine therapies.

The results of this study indicated the potent therapeutic effects of the novel peptide KCK18 against endotoxemia and sepsis and highlighted that cytokine production is a crucial pathogenic mechanism underlying endotoxemia and sepsis. The production of multiple inflammatory cytokines, as observed in endotoxemia and sepsis, is a critical condition frequently referred to as a “cytokine storm” [43]. Naturally occurring microbial infection is the most common cause of a cytokine storm [43]. In addition to disseminated bacterial infection, a cytokine storm can be caused by a disseminated viral infection, such as COVID-19, which is caused by severe acute respiratory syndrome coronavirus 2 [44]. Thus, anticytokine therapies (e.g., the IL-6 receptor antagonists tocilizumab and sarilumab) have been proposed to be incorporated in the clinical treatment of critically ill patients with COVID-19 [45,46]. Since the novel peptide KCF18 can simultaneously inhibit three cytokines, KCF18 may exert significant therapeutic effects on critical conditions that cause a cytokine storm. Additional studies are required before definitive conclusions can be drawn.

This study has some limitations that should be addressed. First, this study investigated only one treatment protocol. Using pharmacokinetic data, we developed a treatment protocol that involved the administration of a loading dose of KCF18 followed by the administration of three supplemental doses of KCF18 every 2 h. Moreover, to more effectively simulate real-world clinical conditions (i.e., patients usually do not seek medical assistance and receive therapy until clinical symptoms and signs are evident), we decided to begin KCF18 treatment after endotoxemia induction (i.e., 2 h after LPS administration). Although our results confirmed the therapeutic effects of the KCF18-based treatment protocol on endotoxemia, whether this treatment protocol is the most effective remains to be elucidated. Second, peptides are rapidly degraded in vivo due to a lack of support [21,47]. Therefore, repeated doses of peptide drugs are required to maintain therapeutic levels. Peptides can be conjugated with polymers to enhance their stability and increase their therapeutic efficacy [47]. However, this study did not employ peptides with polymer conjugation. Thus, we could not evaluate whether polymer conjugation can enhance the therapeutic effects of the KCF18 peptide on endotoxemia. Third, this study investigated the therapeutic effects of KCF18 only within 24 h after endotoxemia induction. Therefore, whether this protocol can exert prolonged therapeutic effects on endotoxemia remains unclear. Fourth, KCF18 could simultaneously inhibit three cytokines. However, whether the current structure of KCF18 is in its most effective form for efficiently inhibiting the three crucial cytokines remains to be elucidated. Fifth, this study employed endotoxemia, i.e., a widely used a monomicrobial sepsis model [29], to facilitate investigation. This model provides a simple and reproducible animal model of sepsis [29]. However, sepsis involves complex biology and pathophysiology [48]. Thus, one may challenge that endotoxemia induced by endotoxin may not be an appropriate model for replicating human sepsis (e.g., Wiggers–Bernard Conference on preclinical sepsis modeling) [49]. In addition to endotoxemia, several other animal models of sepsis have been developed, including models of bacterial injection, implantation of fibrin clots with bacteria inclusion, cecal ligation and puncture, etc. [50]. As this study only employed the model of endotoxemia, the question of whether the novel peptide KCF18 can exert similar beneficial effects in the other sepsis models remains to be elucidated.

## 5. Conclusions

KCF18 peptide can simultaneously inhibit TNF-α, IL-1β, and IL-6 via concurrently blocking bindings of TNF-α, IL-1β, and IL-6 to their cognate receptors in liver tissues in endotoxemic mice. Moreover, KCF18 peptide can alleviate liver injury in mice with endotoxemia. The mechanisms may involve inhibitions of inflammation, oxidation and the cell death processes of necroptosis, pyroptosis, and apoptosis.

## Data Availability

All data are reported in the present study.
